# Antipsychotic-placebo separation on the PANSS-6 subscale as compared to the PANSS-30: a pooled participant-level analysis

**DOI:** 10.1038/s41537-021-00168-x

**Published:** 2021-08-27

**Authors:** Fredrik Hieronymus, Pernille Kølbæk, Christoph U. Correll, Søren D. Østergaard

**Affiliations:** 1 Department of Clinical Medicine, Aarhus University, Aarhus, Denmark; 2 Department of Affective Disorders, Aarhus University Hospital—Psychiatry, Aarhus, Denmark; 3 Institute of Neuroscience and Physiology, University of Gothenburg, Gothenburg, Sweden; 4 Psychosis Research Unit, Aarhus University Hospital—Psychiatry, Aarhus, Denmark; 5 Division of Psychiatry Research, The Zucker Hillside Hospital, Glen Oaks, NY USA; 6 Department of Psychiatry and Molecular Medicine, Hofstra Northwell School of Medicine, Hempstead, NY USA; 7 Department of Child and Adolescent Psychiatry and Psychotherapy, Charité Universitätsmedizin, Berlin, Germany

**Keywords:** Schizophrenia, Biomarkers

## Abstract

In order for measurement-based care to be implemented, there is a need for brief rating instruments that can be administered in a short amount of time, but that are still sufficiently informative. Here, we assessed the drug–placebo sensitivity of the six-item subscale (PANSS-6) of the 30-item Positive and Negative Syndrome Scale (PANSS-30) using a large collection of patient-level data (*n* = 6685) from randomized controlled trials of risperidone and paliperidone. When analyzing the data by study, we found no material difference in mean effect sizes (ES) between the two measures (PANSS-30 ES = 0.45, PANSS-6 ES = 0.44; *p* = 0.642). Stratifying the pooled population according to several putative effect moderators (e.g., age, formulation, dose, or diagnosis) generally yielded no meaningful ES differences between the two measures. Similarly, early improvement (≥20% improvement at week 1) on the PANSS-6 predicted subsequent response (≥40% improvement at endpoint) as well as the analog prediction using PANSS-30. Finally, cross-sectional symptom remission assessed via the PANSS-6 showed very good agreement (sensitivity = 100%, specificity = 98%) with cross-sectional symptom remission defined by the Remission in Schizophrenia Working Group.

## Introduction

The 30-item Positive and Negative Syndrome Scale (PANSS-30)^1^ is the most widely used rating instrument in schizophrenia. While widespread in research settings, it is not readily amenable to routine clinical use because it takes 45–60 min to assess all 30 PANSS items^1^. Clinical practice may therefore be better served by using brief rating instruments that can be completed in a short amount of time, e.g., for routine objective tracking of short-term disease progression or improvement, or for assessing sustained response and remission^2–4^.

One such brief rating instrument is the unidimensional six-item PANSS subscale (PANSS-6). Following up on prior item-level analyses of the PANSS^5,6^, the PANSS-6 was derived as a unidimensional measure of schizophrenia severity via item response theory analyses of the eight-item PANSS-based definition of symptom remission from the Remission in Schizophrenia Working Group^2,7^. The PANSS-6 subscale includes three items measuring positive symptoms (P1 Delusions, P2 Conceptual disorganization, and P3 Hallucinatory behavior) and three items measuring negative symptoms (N1 Blunted affect, N4 Passive/apathetic social withdrawal, and N6 Lack of spontaneity and flow of conversation). The sensitivity of the PANSS-6, when extracted from PANSS-30 assessments, has previously been found to match that of the PANSS-30 as far as antipsychotic–placebo differences^7^ and differences between antipsychotics are concerned^8,9^. The PANSS-6 also has a high rate of agreement with the PANSS-based definition of symptom remission from the Remission in Schizophrenia Working Group^2,8,9^. By using the Simplified Negative and Positive Symptoms Interview (SNAPSI) a PANSS-6 rating can be completed in 15–20 min^10^. Furthermore, PANSS-6 ratings obtained using the SNAPSI have been shown to have good inter-rater reliability^11,12^ and validity when using PANSS-6 ratings obtained via SCI-PANSS of the same patients conducted by independent raters as the reference^10^. Accordingly, PANSS-6 was recently highlighted as an alternative to longer clinician-rated scales in the practice guideline for the treatment of schizophrenia published by the American Psychiatric Association^13^.

In this study, we compared the sensitivity of PANSS-6 and PANSS-30 to the efficacy of antipsychotics in a large collection of patient-level data (*n* = 6685) from 18 acute-phase trials of antipsychotics in schizophrenia and schizoaffective disorder, which had used the PANSS-30 as outcome measure. We aimed to assess if there are conditions under which the PANSS-6 might be less sensitive than the PANSS-30, or conversely, if there are situations in which PANSS-6 may provide an advantage. Thus, we first compared the PANSS-6 and PANSS-30 in terms of their sensitivity to drug–placebo differences for all 18 included trials individually. We then pooled all studies and assessed sensitivity across several putative effect moderators (e.g., time under treatment, baseline severity, drug formulation). We also assessed how well cross-sectional symptom remission defined by the PANSS-6 aligned with the cut-off for symptom remission defined by the Remission in Schizophrenia Working Group^2^, without requiring the 6-month time criterion. Finally, since early symptomatic improvement on the PANSS-30 has been shown to be a strong predictor of subsequent response^14–17^, we also assessed the positive predictive value (PPV) and the negative predictive value (NPV) of early improvement on the PANSS-6 and PANSS-30. We hypothesized that PANSS-6, which can be assessed in much less time than then PANSS-30, would perform on par with PANSS-30, thereby presenting a clinically valid and useful measurement-based care instrument for both clinical care and research purposes.

## Results

### Included studies

In total, 18 placebo-controlled studies with 46 antipsychotic–placebo comparisons were available for inclusion. Of these, nine investigated paliperidone extended release (*n* = 3232), five investigated paliperidone palmitate (*n* = 2085), three investigated risperidone (*n* = 1029), and one investigated risperidone depot (*n* = 336). One study, R076477-SCH-302, included elderly patients only; two studies, R076477-PSZ-3001 and RIS-SCH-302, only included adolescents; and the remaining studies included adults. Ten out of 12 studies investigating per oral (PO) formulations were of 6 weeks duration; the remaining 2 were of 4 weeks duration. Four out of six studies investigating long-acting injectables (LAIs) were of 13 weeks duration; the remaining two were of 12 and 9 weeks duration, respectively. Details of the included studies are displayed in Table 1.

**Table 1:** Details of included studies.

Study	Diagnosis	Treatment length (weeks)	Treatment	*n*	PANSS baseline (SD)	PANSS-6 baseline (SD)	Age (SD)	*n* (%) female
**Paliperidone ER**								
R076477-PSZ-3001	Schizophrenia	6	Paliperidone ER 1.5 mg	54	91.6 (12.5)	20.0 (3.1)	NA^a^	24 (44)
			Paliperidone ER 3 or 6^b^mg	48	90.6 (14.0)	20.0 (4.0)	NA^a^	17 (35)
			Paliperidone ER 6 or 12^b^mg	48	91.7 (13.7)	20.8 (3.4)	NA^a^	14 (29)
			Placebo	51	90.6 (12.1)	21.6 (3.7)	NA^a^	28 (55)
R076477-SCA-3001	Schizoaffective disorder	6	Paliperidone ER 3–6 mg	108	95.7 (13.1)	19.6 (3.8)	37.6 (9.5)	37 (34)
			Paliperidone ER 9–12 mg	99	92.6 (12.5)	18.9 (4.1)	36.5 (10.2)	34 (34)
			Placebo	107	91.6 (12.5)	18.7 (3.9)	36.3 (10.3)	40 (37)
R076477-SCA-3002	Schizoaffective disorder	6	Paliperidone ER 3–12 mg	216	92.1 (13.5)	19.3 (4.0)	37.3 (8.9)	96 (44)
			Placebo	95	91.7 (12.0)	22.9 (4.3)	36.1 (10.5)	29 (37)
R076477-SCH-3015	Schizophrenia	6	Paliperidone ER 6–12 mg	160	102.9 (13.2)	22.4 (4.1)	35.8 (11.7)	53 (33)
			Quetiapine 50–800 mg	159	101.6 (13.5)	22.2 (3.8)	36.9 (10.3)	50 (31)
			Placebo	79	103.7 (15.7)	22.9 (4.3)	36.1 (10.5)	29 (37)
R076477-SCH-302	Schizophrenia	6	Paliperidone 3–12 mg	76	91.8 (9.7)	21.5 (3.8)	70.0 (5.0)	56 (74)
			Placebo	38	94.3 (9.0)	21.4 (3.8)	69.1 (3.4)	27 (71)
R076477-SCH-303	Schizophrenia	6	Olanzapine 10 mg	127	93.0 (10.7)	22.3 (3.6)	35.8 (11.8)	68 (54)
			Paliperidone ER 6 mg	123	94.3 (10.5)	22.4 (3.3)	36.8 (10.7)	62 (50)
			Paliperidone ER 9 mg	122	93.2 (11.9)	22.0 (3.5)	38.0 (11.5)	50 (41)
			Paliperidone ER 12 mg	130	94.5 (11.0)	22.5 (3.5)	35.5 (10.9)	60 (46)
			Placebo	127	94.0 (10.7)	22.0 (3.3)	37.3 (11.3)	61 (48)
R076477-SCH-304	Schizophrenia	6	Olanzapine 10 mg	112	94.6 (12.3)	22.4 (3.3)	40.4 (10.8)	22 (20)
			Paliperidone 6 mg	113	92.4 (12.0)	22.0 (3.7)	42.1 (10.1)	36 (32)
			Paliperidone 12 mg	114	94.0 (11.3)	22.4 (4.0)	41.4 (10.7)	34 (30)
			Placebo	109	93.0 (11.9)	22.3 (3.5)	42.1 (10.9)	23 (21)
R076477-SCH-305	Schizophrenia	6	Olanzapine 10 mg	127	93.4 (12.2)	22.5 (3.6)	36.8 (10.3)	31 (24)
			Paliperidone 3 mg	127	91.3 (12.1)	21.8 (4.0)	36.2 (10.9)	46 (36)
			Paliperidone 9 mg	125	94.3 (13.4)	22.5 (3.6)	36.0 (10.9)	44 (35)
			Paliperidone 15 mg	114	92.6 (12.7)	22.3 (3.6)	37.7 (9.8)	41 (36)
			Placebo	123	93.8 (12.6)	22.3 (3.6)	37.5 (11.2)	37 (30)
R076477-SCH-4012	Schizophrenia	6	Paliperidone 1.5 mg	66	91.9 (12.8)	22.3 (3.7)	41.3 (11.0)	16 (24)
			Paliperidone 6 mg	70	91.9 (12.7)	22.0 (3.6)	40.3 (12.8)	23 (33)
			Placebo	65	92.6 (12.1)	22.0 (3.6)	36.7 (11.6)	18 (28)
**Paliperidone palmitate**								
PALM-JPN-4	Schizophrenia	13	Paliperidone palmitate 75 mg eq.	163	86.0 (14.7)	21.0 (4.5)	45.7 (13.6)	60 (37)
			Placebo	161	83.3 (15.2)	20.1 (4.5)	44.1 (12.4)	80 (49)
R092670-PSY-3003	Schizophrenia	13	Paliperidone palmitate 50 mg eq.	94	90.0 (10.8)	21.1 (3.4)	38.7 (10.5)	28 (30)
			Paliperidone palmitate 100 mg eq.	97	90.5 (11.8)	21.3 (3.7)	38.7 (10.7)	33 (34)
			Paliperidone palmitate 150 mg eq.	30	92.2 (11.7)	21.4 (4.0)	41.0 (11.5)	8 (27)
			Placebo	135	92.3 (12.5)	21.5 (3.6)	40.3 (11.1)	40 (30)
R092670-PSY-3004	Schizophrenia	13	Paliperidone palmitate 25 mg eq.	131	90.5 (12.2)	21.6 (3.7)	40.3 (10.8)	45 (34)
			Paliperidone palmitate 50 mg eq.	127	91.1 (12.0)	21.8 (3.8)	38.5 (11.9)	34 (27)
			Paliperidone palmitate 100 mg eq.	129	90.7 (11.7)	21.8 (3.6)	41.6 (11.0)	46 (36)
			Placebo	124	90.7 (12.3)	21.8 (3.8)	40.3 (11.8)	48 (39)
R092670-PSY-3007	Schizophrenia	13	Paliperidone palmitate 25 mg eq.	160	87.0 (12.1)	19.9 (3.7)	38.7 (10.4)	44 (28)
			Paliperidone palmitate 100 mg eq.	165	86.3 (10.9)	20.1 (3.6)	38.3 (10.5)	55 (33)
			Paliperidone palmitate 150 mg eq.	162	88.5 (11.6)	20.6 (3.7)	39.0 (11.0)	57 (35)
			Placebo	164	86.8 (10.2)	19.7 (3.2)	39.3 (11.1)	55 (34)
R092670-SCH-201	Schizophrenia	9	Paliperidone palmitate 50 mg eq.	78	93.2 (11.0)	21.8 (3.8)	39.0 (10.2)	22 (28)
			Paliperidone palmitate 100 mg eq.	83	92.8 (11.7)	21.7 (4.3)	37.4 (10.3)	29 (35)
			Placebo	82	94.3 (12.4)	22.5 (4.0)	39.2 (10.6)	29 (35)
**Risperidone**								
RIS-INT-3	Schizophrenia	4	Haloperidol 20 mg	87	93.6 (18.8)	22.8 (4.9)	37.6 (9.7)	13 (15)
			Risperidone 2 mg	87	89.2 (19.2)	21.7 (5.6)	38.4 (10.7)	15 (17)
			Risperidone 6 mg	86	94.9 (19.9)	22.8 (5.5)	36.9 (10.4)	15 (17)
			Risperidone 10 mg	85	91.9 (19.3)	22.1 (5.3)	36.3 (9.9)	12 (14)
			Risperidone 16 mg	87	93.4 (18.1)	22.1 (5.2)	37.3 (10.9)	17 (20)
			Placebo	88	92.5 (17.4)	21.9 (4.4)	37.2 (10.5)	14 (16)
RIS-SCH-302	Schizophrenia	6	Risperidone 1–3 mg	54	95.6 (10.8)	21.8 (3.3)	15.7 (1.3)	24 (44)
			Risperidone 4–6 mg	50	93.3 (11.9)	21.4 (3.8)	15.7 (1.3)	14 (28)
			Placebo	53	93.3 (10.4)	21.0 (3.6)	15.5 (1.4)	18 (34)
RIS-USA-72	Schizophrenia	4	Risperidone 4 mg	85	94.3 (11.4)	22.6 (3.6)	38.2 (9.3)	18 (21)
			Risperidone 8 mg	77	93.8 (10.2)	22.6 (3.9)	37.9 (8.7)	14 (18)
			Placebo	83	94.3 (10.9)	21.8 (3.7)	38.2 (11.1)	18 (22)
**Risperidone depot**								
RIS-USA-121	Schizophrenia and schizoaffective disorder	12	Risperidone depot 25 mg	105	81.8 (12.8)	19.2 (4.4)	39.0 (9.8)	36 (34)
			Risperidone depot 50 mg	118	82.5 (14.4)	19.2 (4.8)	37.2 (9.4)	27 (23)
			Risperidone depot 75 mg	113	79.7 (13.9)	18.3 (3.8)	38.5 (10.6)	37 (33)
			Placebo	107	82.4 (14.0)	18.9 (4.2)	38.1 (9.3)	25 (23)

*ER* extended release, SD standard deviation. ^a^Individual ages were not included in the data set. Patients were between 12 and 17 years of age at inclusion. ^b^Dosages varied depending on body weight. All included studies are of acute-phase studies.

### Study-level comparisons between PANSS-30 and PANSS-6

Table 2 details all comparisons between active treatment and placebo. Out of 46 antipsychotic–placebo comparisons, a statistically significant superiority of treatment was found for 38 when the PANSS-30 was used as the effect parameter. Likewise, superiority of treatment was found for 39 pairs when the PANSS-6 was the outcome measure. Seven treatment–placebo comparisons showed no statistically significant separation on either outcome measure and 38 showed statistically significant separation on both outcome measures. The one comparison that differed between outcome measures was the high-dose group (paliperidone extended release 6–12 mg) in Study R076477-PSZ-3001, where the PANSS-6 showed a statistically significant superiority of active treatment (ES 0.51, *p* = .013), while the PANSS-30 did not (ES 0.33; *p* = 0.102). Five drug–placebo comparisons showed an ES difference between the PANSS-6 and PANSS-30 above 0.10 (three favoring PANSS-6). Endpoint item scores for these five comparisons are provided in Supplementary Tables 1–5. When analyzing all active treatments in each trial as a group, the arithmetic mean effect size across trials was 0.45 for the PANSS-30 and 0.44 for the PANSS-6; with a non-significant difference in effect size between the two scales of 0.0061 (SEM 0.0130; *p* = 0.642). Effect sizes were numerically larger for PANSS-30 than for PANSS-6 in 11 out of 18 trials (*p* = 0.346).

**Table 2:** Antipsychotic–placebo differences as measured by PANSS-30 and PANSS-6 stratified by study and dose.

Study	Antipsychotic	MD PANSS-30 (SEM)	ES (95% CI)	*P*	MD PANSS-6 (SEM)	ES (95% CI)	*P*
**Paliperidone extended release**							
R076477-PSZ-3001	PER 1.5 mg	1.9 (3.3)	0.11 (−0.27 to 0.49)	0.5734	0.7 (0.9)	0.16 (−0.22 to 0.55)	0.4057
	PER 3 or 6 mg	9.5 (3.4)	0.56 (0.17–0.96)	**0.0057**	2.4 (0.9)	0.56 (0.17–0.96)	**0.0057**
	PER 6 or 12 mg	5.6 (3.4)	0.33 (−0.06 to 0.73)	0.1017	2.2 (0.9)	0.51 (0.11–0.90)	**0.0134**
R076477-SCA-3001	PER 3–6 mg	3.5 (2.7)	0.18 (−0.09 to 0.45)	0.1941	0.7 (0.6)	0.15 (−0.11 to 0.42)	0.2618
	PER 9–12 mg	8.5 (2.7)	0.44 (0.16–0.71)	**0.0019**	1.7 (0.6)	0.40 (0.13–0.68)	**0.0041**
R076477-SCA-3002	PER 3–12 mg	7.7 (2.2)	0.43 (0.19–0.67)	**0.0006**	1.8 (0.6)	0.40 (0.16–0.64)	**0.0012**
R076477-SCH-3015	PER 6–12 mg	6.2 (2.6)	0.32 (0.05–0.59)	**0.0191**	1.6 (0.6)	0.34 (0.07–0.61)	**0.0135**
	QTP 50 to 800 mg	2.5 (2.6)	0.13 (−0.14–0.40)	0.3383	0.9 (0.6)	0.20 (−0.07 to 0.47)	0.1489
R076477-SCH-302	PER 3 to 12 mg	5.7 (2.8)	0.41 (0.02–0.80)	**0.0414**	1.7 (0.8)	0.43 (0.04–0.82)	**0.0310**
R076477-SCH-303	OLZ 10 mg	16.2 (2.6)	0.80 (0.55–1.04)	**<0.0001**	4.0 (0.6)	0.80 (0.55–1.04)	**<0.0001**
	PER 6 mg	13.9 (2.6)	0.68 (0.43–0.93)	**<0.0001**	3.6 (0.6)	0.72 (0.47–0.97)	**<0.0001**
	PER 9 mg	13.3 (2.6)	0.66 (0.41–0.91)	**<0.0001**	3.1 (0.6)	0.62 (0.38–0.87)	**<0.0001**
	PER 12 mg	18.6 (2.5)	0.91 (0.67–1.16)	**<0.0001**	4.3 (0.6)	0.86 (0.61–1.10)	**<0.0001**
R076477-SCH-304	OLZ 10 mg	9.2 (2.6)	0.47 (0.21–0.73)	**0.0005**	2.1 (0.7)	0.42 (0.16–0.69)	**0.0018**
	PER 6 mg	8.1 (2.6)	0.41 (0.15–0.68)	**0.0022**	2.1 (0.7)	0.43 (0.16–0.69)	**0.0016**
	PER 12 mg	9.9 (2.6)	0.50 (0.24–0.77)	**0.0002**	2.4 (0.7)	0.49 (0.23–0.75)	**0.0003**
R076477-SCH-305	OLZ 10 mg	15.2 (2.5)	0.78 (0.53–1.03)	**<0.0001**	3.7 (0.6)	0.75 (0.50–1.00)	**<0.0001**
	PER 3 mg	12.3 (2.5)	0.63 (0.38–0.88)	**<0.0001**	2.8 (0.6)	0.57 (0.32–0.81)	**<0.0001**
	PER 9 mg	13.0 (2.5)	0.66 (0.41–0.91)	**<0.0001**	2.9 (0.6)	0.59 (0.34–0.84)	**<0.0001**
	PER 15 mg	16.6 (2.5)	0.85 (0.59–1.10)	**<0.0001**	3.7 (0.6)	0.75 (0.50–1.01)	**<0.0001**
R076477-SCH-4012	PER 1.5 mg	−2.7 (4.1)	−0.12 (−0.46 to 0.23)	0.5087	−0.2 (1.0)	−0.03 (−0.37 to 0.31)	0.8609
	PER 6 mg	1.9 (4.1)	0.08 (−0.26 to 0.42)	0.6426	1.2 (1.0)	0.20 (−0.14 to 0.54)	0.2467
**Paliperidone palmitate**							
PALM-JPN-4	PER DPT 75 mg eq.	9.6 (2.2)	0.49 (0.27–0.71)	**<0.0001**	2.2 (0.5)	0.44 (0.23–0.66)	**0.0001**
R092670-PSY-3003	PER DPT 50 mg eq.	3.8 (2.6)	0.20 (−0.07 to 0.46)	0.1477	1.0 (0.7)	0.20 (−0.07 to 0.46)	0.1430
	PER DPT 100 mg eq.	6.2 (2.6)	0.32 (0.06–0.58)	**0.0189**	2.0 (0.7)	0.41 (0.15–0.67)	**0.0025**
	PER DPT 150 mg eq.	1.9 (3.9)	0.10 (−0.30 to 0.49)	0.6257	0.9 (1.0)	0.18 (−0.22 to 0.57)	0.3754
R092670-PSY-3004	PER DPT 25 mg eq.	6.5 (2.5)	0.32 (0.08–0.57)	**0.0103**	1.9 (0.6)	0.38 (0.13–0.63)	**0.0026**
	PER DPT 50 mg eq.	6.6 (2.6)	0.33 (0.08–0.58)	**0.0103**	1.5 (0.6)	0.30 (0.05–0.55)	**0.0174**
	PER DPT 100 mg eq.	9.5 (2.6)	0.47 (0.23–0.72)	**0.0002**	1.9 (0.6)	0.38 (0.13–0.63)	**0.0026**
R092670-PSY-3007	PER DPT 25 mg eq.	4.7 (2.0)	0.26 (0.05–0.48)	**0.0181**	1.0 (0.5)	0.22 (0.01–0.44)	**0.0443**
	PER DPT 100 mg eq.	8.9 (2.0)	0.49 (0.27–0.71)	**<0.0001**	1.8 (0.5)	0.40 (0.18–0.61)	**0.0004**
	PER DPT 150 mg eq.	9.7 (2.0)	0.53 (0.32–0.75)	**<0.0001**	2.2 (0.5)	0.49 (0.27–0.71)	**<0.0001**
R092670-SCH-201	PER DPT 50 mg eq.	14.1 (3.4)	0.66 (0.35–0.97)	**<0.0001**	2.8 (0.9)	0.52 (0.21–0.83)	**0.0013**
	PER DPT 100 mg eq.	17.7 (3.4)	0.82 (0.52–1.13)	**<0.0001**	4.0 (0.8)	0.74 (0.43–1.05)	**<0.0001**
**Risperidone**							
RIS-INT-3	HPL 20 mg	8.1 (3.3)	0.37 (0.07–0.66)	**0.0160**	1.9 (0.8)	0.35 (0.06–0.65)	**0.0205**
	RIS 2 mg	9.2 (3.3)	0.42 (0.12–0.71)	**0.0062**	2.3 (0.8)	0.43 (0.13–0.73)	**0.0049**
	RIS 6 mg	20.7 (3.4)	0.94 (0.64–1.23)	**<0.0001**	4.4 (0.8)	0.83 (0.53–1.13)	**<0.0001**
	RIS 12 mg	13.2 (3.4)	0.60 (0.30–0.90)	**0.0001**	3.3 (0.8)	0.61 (0.32–0.91)	**0.0001**
	RIS 16 mg	17.2 (3.3)	0.78 (0.48–1.07)	**<0.0001**	3.6 (0.8)	0.68 (0.38–0.98)	**<0.0001**
RIS-SCH-302	RIS 1 to 3 mg	11.2 (3.3)	0.66 (0.28–1.04)	**0.0009**	2.9 (0.8)	0.70 (0.32–1.08)	**0.0004**
	RIS 4 to 6 mg	12.2 (3.4)	0.72 (0.33–1.10)	**0.0004**	2.8 (0.8)	0.68 (0.30–1.07)	**0.0007**
RIS-USA-72	RIS 4 mg	6.8 (3.2)	0.33 (0.02–0.63)	**0.0354**	1.8 (0.8)	0.33 (0.03–0.64)	**0.0356**
	RIS 8 mg	9.7 (3.3)	0.47 (0.16–0.78)	**0.0035**	2.0 (0.9)	0.38 (0.06–0.69)	**0.0190**
**Risperidone depot**							
RIS-USA-121	RIS DPT 25 mg	8.7 (2.3)	0.52 (0.25–0.79)	**0.0002**	2.8 (0.6)	0.62 (0.35–0.89)	**<0.0001**
	RIS DPT 50 mg	9.2 (2.2)	0.55 (0.29–0.81)	**<0.0001**	2.7 (0.6)	0.61 (0.35–0.87)	**<0.0001**
	RIS DPT 75 mg	6.8 (2.3)	0.41 (0.14–0.67)	**0.0029**	1.9 (0.6)	0.43 (0.17–0.70)	**0.0015**

Significant contrasts are denoted in bold. *DPT* depot formulation, *ES* effect size, *HPL* haloperidol, *MD* mean difference, *OLZ* olanzapine, *PER* paliperidone extended release, *QTP* quetiapine, *RIS* risperidone, *SEM* standard error of the mean.

### Pooled comparisons between PANSS-30 and PANSS-6

Table 3 details the results of pooled analyses stratified for putative effect moderators. The overall pooled effect size (0.46 for PANSS-30 and 0.45 for PANSS-6) was similar to the arithmetic mean effect size. In most analyses (13 out of 18), the PANSS-30 yielded numerically larger effect size. With the exception of the analyses in old age (≥65) individuals, where PANSS-6 had an effect size 0.09 units larger than PANSS-30, effect size differences did not exceed 0.03 in favor of either outcome measure.

**Table 3:** Pooled antipsychotic–placebo differences as measured by PANSS-30 and PANSS-6 stratified on putative effect moderators.

Analysis	*n* Antipsychotic	*n* Placebo	MD PANSS-30 (SEM)	ES (95% CI)	*P*	MD PANSS-6 (SEM)	ES (95% CI)	*P*
Full population (LOCF until endpoint)	4890	1795	9.1 (0.6)	0.46 (0.41–0.51)	<0.0001	2.2 (0.1)	0.45 (0.40–0.50)	<0.0001
Other time points								
LOCF until week 2	4890	1795	4.4 (0.4)	0.30 (0.25–0.35)	<0.0001	1.0 (0.1)	0.27 (0.22–0.32)	<0.0001
LOCF until week 4	4890	1795	6.7 (0.5)	0.38 (0.33–0.43)	<0.0001	1.5 (0.1)	0.35 (0.30–0.40)	<0.0001
LOCF until week 6	4890	1795	8.2 (0.5)	0.43 (0.38–0.48)	<0.0001	1.9 (0.1)	0.40 (0.35–0.45)	<0.0001
Formulation								
LAI	1756	777	8.5 (0.8)	0.45 (0.37–0.53)	<0.0001	2.0 (0.2)	0.42 (0.34–0.50)	<0.0001
PO	3134	1018	9.5 (0.7)	0.48 (0.41–0.55)	<0.0001	2.3 (0.2)	0.47 (0.40–0.54)	<0.0001
Diagnosis								
Schizoaffective disorder	453	211	7.1 (1.6)	0.38 (0.22–0.54)	<0.0001	1.6 (0.4)	0.37 (0.21–0.53)	<0.0001
Schizophrenia	4437	1584	9.4 (0.5)	0.47 (0.41–0.53)	<0.0001	2.3 (0.2)	0.46 (0.40–0.52)	<0.0001
Baseline severity^a^								
PANSS-30 ≤ 90	2366	909	7.7 (0.7)	0.42 (0.34–0.50)	<0.0001	1.8 (0.2)	0.40 (0.32–0.48)	<0.0001
PANSS-30 > 90	2520	883	10.4 (0.8)	0.50 (0.42–0.58)	<0.0001	2.5 (0.2)	0.49 (0.41–0.57)	<0.0001
PANSS-6 ≤ 20	2062	816	8.0 (0.8)	0.44 (0.36–0.52)	<0.0001	1.9 (0.2)	0.45 (0.37–0.53)	<0.0001
PANSS-6 > 20	2825	977	9.9 (0.8)	0.48 (0.41–0.55)	<0.0001	2.4 (0.2)	0.45 (0.38–0.52)	<0.0001
Dosage								
Lower doses (within-trial)	1383	1420	7.7 (0.8)	0.38 (0.31–0.45)	<0.0001	1.9 (0.2)	0.38 (0.31–0.45)	<0.0001
Higher doses (within-trial)	1306	1420	11.2 (0.8)	0.56 (0.48–0.64)	<0.0001	2.6 (0.2)	0.53 (0.45–0.61)	<0.0001
Age^b^								
Adolescents (<18)	250	106	10.2 (2.2)	0.55 (0.32–0.78)	<0.0001	2.6 (0.5)	0.58 (0.35–0.81)	<0.0001
Adults (18–65)	4450	1610	9.8 (0.6)	0.47 (0.41–0.53)	<0.0001	2.3 (0.2)	0.45 (0.39–0.51)	<0.0001
Old adults (≥65)	113	55	6.5 (2.6)	0.42 (0.10–0.74)	0.0118	2.2 (0.7)	0.51 (0.19–0.83)	0.0024

*ES* effect size, *LOCF* last observation carried forward, *MD* mean difference.

^a^Complete information on baseline severity was not available for all individuals. ^b^Individual age was not available for all patients.

### Comparison of symptomatic remission between PANSS-6 and PANSS-8

According to the PANSS-8 symptom remission criteria, 21.6% of placebo-treated participants and 33.8% of actively treated participants reached cross-sectional remission (meeting the PANSS-8 symptom remission criteria at the last available visit). Analyses yielded an additional 1.1% remitted patients on placebo and 1.5% on active treatment when only the PANSS-6 criteria were applied. Among these PANSS-6 remitters, 21 individuals scored 4 points on G5 Mannerisms and posturing, but were otherwise in PANSS-8-defined remission; 67 scored 4 points and 2 scored 5 points on G9 Unusual thought content, but were otherwise in PANSS-8-defined remission, and 4 individuals scored 4 points on both G5 Mannerisms and posturing and G9 Unusual though content. There were thus 94 ‘false positives’ (i.e., remitters on PANSS-6 but not on PANSS-8), yielding a specificity of 98.0%. Patients in PANSS-6 remission but not in PANSS-8 remission (mean PANSS-30: 69.4) had significantly (*p* < 0.0001) higher PANSS-30 scores than patients in PANSS-8 remission (mean PANSS-30: 56.9) but significantly (*p*<0.0001) lower PANSS-30 scores than patients who were not in remission according to either criteria (mean PANSS-30: 88.2).

### Comparisons of PPV and NPV for PANSS-30 and PANSS-6

**Fig. 1:**
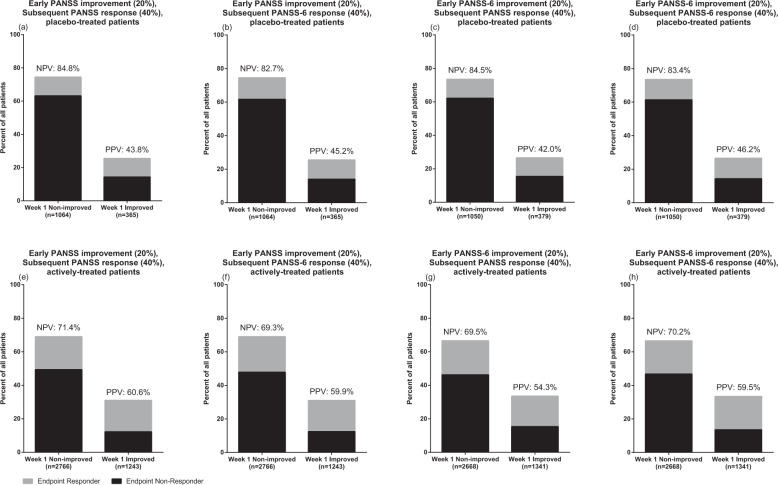
Positive and negative predictive value of early improvement on subsequent response. NPV negative predictive value, PPV positive predictive value.

Figure 1a–h details the PPV and NPV of early response (≥20% decrease in PANSS-6/PANSS-30 at week 1) as it pertains to ultimate response (≥40% decrease in PANSS-6/PANSS-30 at the last available observation). While the NPV was higher for placebo-treated patients (82.7–84.8%; Fig. 1a–d) than for trial participants receiving active treatment (69.3–71.4%; Fig. 1e–h), the PPV was higher for actively treated participants (54.3–60.6%; Fig. 1a–d) than for placebo-treated patients (42.0–46.2%; Fig. 1e–h). There were no major differences in PPV or NPV either across or within PANSS scales, i.e., early improvement on PANSS-6 predicted subsequent response on both the PANSS-6 and PANSS-30 with comparable accuracy, and early improvement on the PANSS-30 likewise predicted subsequent response on both the PANSS-6 and PANSS-30 with comparable accuracy.

### Effect sizes for individual PANSS items

Table 4 contains the results from the post hoc analysis of effect sizes for individual PANSS items. Most effect sizes (26/30) were in the range of 0.20–0.40. The lowest effect size was observed for item G7 Motor retardation (ES 0.11), and the highest effect sizes were seen for the two positive symptoms P2 Conceptual disorganization and P6 Suspiciousness/persecution (ES 0.40 for both items). The impact of adding a specific item to the PANSS-6 largely mirrored effect sizes for the individual items. The largest improvement in subscale effect size (4.6%) was seen for item P7 Hostility, and the largest decline (−4.1%) was seen for item G7 Motor retardation.

**Table 4:** Antipsychotic–placebo differences on individual PANSS-30 items (pooled population).

	Baseline score		Endpoint score				If included in PANSS-6	
Item	Placebo	Antipsychotic	Placebo	Antipsychotic	ES (95% CI)	*P*	ES	% ES-diff
*P1 Delusions*	4.1	4.1	3.6	3.1	0.38 (0.33–0.43)	<0.0001	NA	NA
*P2 Conceptual disorganization*	3.5	3.6	3.3	2.9	0.40 (0.35–0.45)	<0.0001	NA	NA
*P3 Hallucinatory behavior*	3.7	3.7	3.2	2.7	0.36 (0.31–0.41)	<0.0001	NA	NA
P4 Excitement	2.9	2.9	2.7	2.3	0.33 (0.28–0.38)	<0.0001	0.46	3.0%
P5 Grandiosity	2.4	2.4	2.2	2.0	0.21 (0.16–0.26)	<0.0001	0.45	0.8%
P6 Suspiciousness/persecution	3.9	3.9	3.4	2.9	0.40 (0.35–0.45)	<0.0001	0.46	3.1%
P7 Hostility	2.5	2.5	2.4	2.0	0.38 (0.33–0.43)	<0.0001	0.47	4.6%
*N1 Blunted affect*	3.3	3.4	3.1	2.9	0.21 (0.16–0.26)	<0.0001	NA	NA
N2 Emotional withdrawal	3.5	3.6	3.3	3.0	0.28 (0.23–0.33)	<0.0001	0.44	−1.9%
N3 Poor rapport	2.9	3.0	2.8	2.5	0.35 (0.30–0.40)	<0.0001	0.46	1.5%
*N4 Passive/apathetic, social withdrawal*	3.7	3.6	3.3	3.0	0.27 (0.22–0.32)	<0.0001	NA	NA
N5 Difficulty in abstract thinking	3.8	3.8	3.6	3.4	0.23 (0.18–0.28)	<0.0001	0.44	−1.6%
*N6 Lack of spontaneity and flow of conversation*	3.0	3.1	2.9	2.6	0.28 (0.23–0.33)	<0.0001	NA	NA
N7 Stereotyped thinking	3.1	3.1	3.0	2.7	0.26 (0.21–0.31)	<0.0001	0.45	−0.8%
G1 Somatic concern	2.5	2.6	2.4	2.2	0.15 (0.10–0.20)	<0.0001	0.44	−2.4%
G2 Anxiety	3.2	3.2	2.9	2.6	0.28 (0.23–0.33)	<0.0001	0.45	0.5%
G3 Guilt feelings	2.3	2.2	1.9	1.8	0.18 (0.13–0.23)	<0.0001	0.45	−0.1%
G4 Tension	3.1	3.1	2.8	2.4	0.32 (0.27–0.37)	<0.0001	0.46	1.8%
G5 Mannerisms and posturing	2.4	2.4	2.3	2.09	0.22 (0.17–0.27)	<0.0001	0.45	−0.7%
G6 Depression	2.7	2.6	2.4	2.2	0.18 (0.13–0.23)	<0.0001	0.44	−1.6%
G7 Motor retardation	2.3	2.3	2.1	2.0	0.11 (0.06–0.16)	<0.0001	0.43	−4.1%
G8 Uncooperativeness	2.3	2.3	2.4	2.0	0.32 (0.27–0.37)	<0.0001	0.46	1.7%
G9 Unusual thought content	3.5	3.6	3.2	2.9	0.30 (0.25–0.35)	<0.0001	0.45	−0.2%
G10 Disorientation	2.1	2.1	2.0	1.8	0.23 (0.18–0.28)	<0.0001	0.45	1.1%
G11 Poor attention	2.9	2.9	2.8	2.5	0.31 (0.26–0.36)	<0.0001	0.45	0.8%
G12 Lack of judgment and insight	3.7	3.6	3.5	3.2	0.28 (0.23–0.33)	<0.0001	0.45	0.1%
G13 Disturbance of volition	3.0	3.0	2.8	2.5	0.25 (0.20–0.30)	<0.0001	0.45	−0.9%
G14 Poor impulse control	2.6	2.6	2.5	2.1	0.31 (0.26–0.36)	<0.0001	0.46	2.6%
G15 Preoccupation	3.3	3.4	3.2	2.8	0.34 (0.29–0.39)	<0.0001	0.46	1.6%
G16 Active social avoidance	3.3	3.4	3.1	2.8	0.31 (0.26–0.36)	<0.0001	0.45	0.3%

Italicized items (P1-3, N1, N4, and N6) are included in the PANSS-6 subscale. *ES* effect size.

## Discussion

The main finding of this study is that the PANSS-6 and the PANSS-30 have comparable sensitivity to antipsychotic efficacy across a range of putative effect moderators. This finding was evidenced by a negligible difference in mean effect sizes when all 18 included trials were analyzed individually; likewise, subgroup analyses showed no effect size differences exceeding 0.03, with the exception of the old age (≥65) subgroup where PANSS-6 showed an ES 0.09 units larger than PANSS-30. Similarly, agreement between cross-sectional symptom remission as defined by PANSS-6 and by the eight-item definition suggested by the Remission in Schizophrenia Working Group^2^ was very high. Moreover, with regard to prediction of subsequent response via early improvement, the PPV and NPV were comparable between the PANSS-6 and the PANSS-30, both within and across outcome scales. That PANSS-6 is equally sensitive to the PANSS-30 with regard to the efficacy of antipsychotics is in line with results from prior studies on both the efficacy^7^ and effectiveness of antipsychotics in the treatment of schizophrenia^9,10^.

While, based on these and previous results, PANSS-6 seems to be an adequately sensitive instrument for tracking core schizophrenia severity, it should be emphasized that PANSS-6 ratings might need to be accompanied by ratings on measures of other constructs that are relevant in relation to the care of individuals with schizophrenia, e.g., depression, anxiety, cognition, agitation/aggression, medication side effects, level of functioning and quality of life^18–22^, and that such considerations will depend on the research questions and areas of individual need being addressed.

With regard to prediction of subsequent response via early improvement, the PPV and NPV of the PANSS-6 were comparable to those of the PANSS-30, both with respect to longitudinal prediction based on the same measure and in comparisons across time and between the PANSS-6 and PANSS-30 (Fig. 1). These results replicate previous findings showing that early improvement on the PANSS-30, as well as on other schizophrenia rating scales^14–17^, is a strong predictor of subsequent response. The fact that this relationship holds also for PANSS-6 is of obvious importance if the PANSS-6 - which does not take as much time to complete as the PANSS-30 - is to be used to reduce contact time in clinical trials that may contribute to an observed inflated placebo response^23^, or to inform personalized treatment in clinical care settings.

The high rate of agreement (100% sensitivity, 98.0% specificity) between cross-sectional symptom remission as defined by the PANSS-8 and PANSS-6 is partly by design since all patients meeting the PANSS-8 criteria also qualify for remission according to the PANSS-6, thus yielding perfect sensitivity. Patients who had remitted according to PANSS-6, but not PANSS-8, had significantly higher PANSS-30 symptom scores than PANSS-8 remitters (69.4 vs 56.9) but significantly lower symptom scores than non-remitters to the PANSS-8 definition (69.4 vs 88.2). This finding suggests that the small fraction of additional PANSS-6 remitters may differ from the larger group of PANSS-8 remitters.

Most individual PANSS items (26 out of 30) had effect sizes in the range of 0.20–0.40. As expected due to the predominant efficacy of currently available antipsychotics for positive rather than negative symptoms^24,25^, positive symptoms were, on average, those that separated most clearly from placebo (Table 4). This contrasts to similar analyses of patients with major depression where much larger disparities in individual-item effect sizes have been reported for the Hamilton Depression Rating Scale^26^. Notably, the two PANSS items with the lowest effect sizes were G7 Motor retardation (ES 0.11) and G1 Somatic concern (ES 0.15), which could reflect that specific side effects of antipsychotics worked against a general improvement in the underlying condition^27,28^, as has been suggested for depression—i.e., that some side effects (e.g., sedation, dystonia, arthralgia, nausea) are mistaken for psychopathology as measured by G7 Motor retardation and G1 Somatic concern^29–31^. Another factor that may contribute to the low drug–placebo separation for these items is the comparatively low baseline scores (2.30 for G7 and 2.54 for G1) in combination with the fact that these symptoms showed some improvement also on placebo (endpoint scores on placebo: 2.10 and 2.35, respectively). Taken together, this leaves little room for a true drug effect to be detected.

This study has a number of limitations. First, although we included a large number of trials and participants, only few trials included certain subgroups, e.g., trials of schizoaffective disorder and trials focusing on adolescents or older adults. The power to detect differences in sensitivity between the PANSS-6 and the PANSS-30 in these subgroups was hence insufficient, and the results should be interpreted with caution. Second, the results stem from data obtained through randomized clinical trials, and it remains to be investigated to what degree these results will generalize to clinical settings. Third, PANSS-6 ratings were derived from the full PANSS-30 ratings; however, PANSS-6 ratings obtained using the SNAPSI may not correspond to those observed when conducting the full SCI-PANSS. Ideally, one should compare data from different raters independently scoring the PANSS-6 and PANSS-30 in the same patients, at the same time. In lieu of such data, analyses extracting PANSS-6 scores from full PANSS-30 ratings are the best alternative. Notably, we recently conducted a study comparing dedicated PANSS-6 assessments via the SNAPSI and PANSS-6 assessments as part of the full PANSS-30 ratings obtained by independent raters and found good agreement across the two methods of obtaining PANSS-6 ratings^11^. Finally, from an implementation perspective, it should be noted that, while the SNAPSI is freely available for non-commercial clinical and research purposes (https://www.medavante-prophase.com/welcome-to-snapsi/), use of the PANSS-30 and its subscales (including the PANSS-6) requires a license agreement with the copyright holder (Multi-Health Systems) and is associated with a fee.

To summarize, in this large-scale patient-level analysis, the sensitivity of the PANSS-6 to antipsychotic efficacy was comparable to that of the PANSS-30 across a range of different tests and putative effect moderators. These findings add to a growing literature indicating that the PANSS-6 can be used to adequately monitor the severity of core schizophrenia symptomatology over time^7–13^. Given its brevity, the PANSS-6 may facilitate the implementation of objective tracking of core schizophrenia severity in the clinic and contribute to making future clinical trials of treatments for schizophrenia less costly and resource intensive.

## Methods

### Data acquisition

We requested patient-level data for all industry-sponsored, acute-phase, placebo-controlled trials of risperidone and paliperidone via the Yale Open Data Access (YODA) project^32^. Remote access to patient-level data was provided by Johnson & Johnson and YODA for all 19 requested studies. One study (RIS-USA-1/Study 201) used the Brief Psychiatric Rating Scale (BPRS) instead of the PANSS and could hence not be included in the analyses. In order to verify the accuracy of the data, we compared our results to those from study reports provided by YODA and with those available in public reports from the United States Food and Drug Administration^33–39^, the European Medicines Agency^40^, and ClinicalTrials.gov^41–43^.

### Analyses and statistics

Individual antipsychotics and doses were first analyzed by trial using analysis of covariance (ANCOVA). Analyses were performed on the intention-to-treat population using last observation carried forward methodology up until the last scheduled evaluation for each trial. The efficacy of all included treatment arms was assessed using both the PANSS-30 and PANSS-6. The models included a fixed factor for treatment and baseline score on the corresponding outcome measure as a covariate. Effect size differences between the PANSS-6 and PANSS-30 were assessed using paired samples *t*-test, and the rate at which effect sizes favored either outcome measure over the other was assessed using the one-sample chi-squared test with both outcomes expected to occur with equal frequency. In order to not include placebo-treated participants more than once (i.e., since a trial may have had several active treatment arms), the two latter analyses were conducted with all patients receiving active treatment analyzed together in each trial.

We then pooled all available studies and conducted analyses stratified by putative effect moderators. The model specifications for the pooled analyses were analogous to those used for the analyses of individual studies with the addition of a fixed factor for study (with one exception, see below). The assessed effect moderators were earlier endpoints (weeks 2, 4, and 6), drug formulation (LAI, or PO), diagnosis (schizophrenia vs. schizoaffective disorder), baseline severity (PANSS-30/PANSS-6 at or below median vs. above median), dose, and age group (adolescents, adults, older adults). For the dose analyses, we included all trials investigating at least two different doses of one active treatment and included the arm with the lowest given dose in the ‘low-dose’ group and the arm with the highest given dose in the ‘high-dose’ group. For the age group analyses, we excluded the study factor since some studies included very few patients belonging to a specific age group.

We then assessed endpoint symptom remission in the pooled population (i.e., a cross-sectional definition of remission not requiring the 6-month time criterion)^2^. We did so by contrasting PANSS-6-defined remission (defined as a score of ≤3 on all PANSS-6 items, range 1–7) with the eight-item definition (defined as a score of ≤3 on all PANSS-6 items as well as on G5 Mannerisms and posturing and G9 Unusual thought content, PANSS-8) suggested by the Remission in Schizophrenia Working Group^2^. Due to the overlap between the criteria, all patients in remission according to the PANSS-8 criteria were by definition also in PANSS-6-defined remission. We thus focused on those additional patients found to be in remission only according to the PANSS-6. We detailed their scores on the two additional items in the PANSS-8 and assessed (via independent samples *t*-tests) whether their PANSS-30 scores were different from those of patients who were in symptom remission according to the PANSS-8 definition, and from those of patients who were not in remission according to either definition, respectively^2^.

We then assessed the PPV and NPV of early improvement (defined as a ≥20% reduction in PANSS-6 or PANSS-30 at the week 1 evaluation) on subsequent response (defined as a ≥40% reduction in PANSS-6 or PANSS-30 at the endpoint evaluation). These assessments were performed both within scales (e.g., early improvement in PANSS-6 predicting PANSS-6 response) and across scales (e.g., early improvement in PANSS-30 predicting PANSS-6 response). Analyses were stratified by treatment (placebo or active treatment). For patients to be included in the analyses, they needed to have an evaluation during week 1 and at least one subsequent evaluation. The last available scheduled evaluation was used as the endpoint evaluation. In order for percentage differences to make intuitive sense, PANSS scores were rescaled from 1 to 7 to 0 to 6 (i.e., so that a patient with no PANSS-30-measured symptoms would score 0 rather than 30) for this analysis.

Finally, based on the observation that the pooled effect sizes obtained with the PANSS-6 and PANSS-30 were almost identical, but slightly higher for the PANSS-30, we conducted the following post hoc analyses. First, we calculated effect sizes for all individual PANSS-30 items. Subsequently, we analyzed how the drug–placebo sensitivity of the PANSS-6 would be impacted by including each of the 24 PANSS-30 items not included in the PANSS-6 (“add-one-in” analysis). The models used for these analyses were identical to those described above but with the outcome parameter being the item in question or that item plus PANSS-6, with the baseline score on the respective outcome parameter being included as a covariate.

All analyses were conducted using R version 3.6.1. Two-sided *p* values <0.05 were considered statistically significant. Due to substantial overlap between outcomes (the PANSS-6 items are nested within the PANSS-30), populations (individual trials are nested within the pooled population), and subgroups (e.g., participants with low scores on the PANSS-6 also tend to have low scores on the PANSS-30), correction for multiple testing was not performed.

### Ethics

The data used for this study consist of de-identified patient-level data from previously conducted clinical trials. Secondary analyses of de-identified data does not fall under the purview of ethical committees in the jurisdiction where the research was carried out.

### Reporting summary

Further information on research design is available in the Nature Research Reporting Summary linked to this article.

## Supplementary information

Reporting summary

Supplementary Tables

## Data Availability

The data used in this article can be requested from the Yale Open Data Access website^32^.

## Code availability

No non-standard code or algorithms were used in the generation of the results in this article.
