# Supplementary material for: Antipsychotic-placebo separation on the PANSS-6 subscale as compared to the PANSS-30: a pooled participant-level analysis
Source: NPJ Schizophr. 2021 Aug 27;7:41. doi: 10.1038/s41537-021-00168-x (PMC8397783; doi:10.1038/s41537-021-00168-x)
Supplement: Supplementary file 2 — Supplementary Tables [file 41537_2021_168_MOESM2_ESM.docx]

**Supplementary Table 1.** Study R076477-PSZ-3001, paliperidone ER 6-12mg (n=48) vs placebo (n=51). Effect sizes for individual items.

|  | **Baseline score** | | **Endpoint score** | |  |  |
| --- | --- | --- | --- | --- | --- | --- |
| **Item** | **Placebo** | **Active** | **Placebo** | **Active** | **ES** | **p** |
| P1 Delusions | 3.67 | 3.60 | 3.04 | 2.58 | 0.47 | **.0250** |
| P2 Conceptual disorganization | 3.16 | 3.48 | 3.21 | 2.71 | 0.51 | **.0125** |
| P3 Hallucinatory behaviour | 3.12 | 3.48 | 2.72 | 2.15 | 0.57 | **.0207** |
| P4 Excitement | 2.90 | 3.02 | 2.72 | 2.24 | 0.48 | **.0427** |
| P5 Grandiosity | 2.45 | 2.23 | 2.04 | 1.81 | 0.22 | .2069 |
| P6 Suspiciousness/persecution | 3.75 | 3.40 | 2.93 | 2.59 | 0.34 | .0877 |
| P7 Hostility | 2.88 | 2.71 | 2.61 | 2.25 | 0.37 | .1169 |
| N1 Blunted affect | 3.25 | 3.63 | 3.17 | 3.05 | 0.11 | .5311 |
| N2 Emotional withdrawal | 3.49 | 3.77 | 3.31 | 3.26 | 0.05 | .8050 |
| N3 Poor rapport | 3.24 | 3.31 | 3.23 | 3.01 | 0.22 | .2829 |
| N4 Passive/apathetic, social withdrawal | 3.65 | 3.77 | 3.31 | 3.34 | -0.03 | .8789 |
| N5 Difficulty in abstract thinking | 3.59 | 3.54 | 3.49 | 3.27 | 0.22 | .2051 |
| N6 Lack of spontaneity and flow of conversation | 3.20 | 3.65 | 3.27 | 2.98 | 0.29 | .1808 |
| N7 Stereotyped thinking | 2.98 | 2.98 | 2.86 | 2.71 | 0.15 | .3991 |
| G1 Somatic concern | 2.29 | 2.15 | 2.02 | 1.97 | 0.05 | .7430 |
| G2 Anxiety | 2.86 | 2.83 | 2.48 | 2.26 | 0.23 | .1984 |
| G3 Guilt feelings | 1.80 | 1.94 | 1.72 | 1.59 | 0.12 | .3999 |
| G4 Tension | 2.78 | 2.71 | 2.50 | 2.22 | 0.27 | .1036 |
| G5 Mannerisms and posturing | 2.71 | 3.08 | 2.54 | 2.28 | 0.25 | .2000 |
| G6 Depression | 2.25 | 2.25 | 1.94 | 1.86 | 0.08 | .6078 |
| G7 Motor retardation | 2.59 | 2.58 | 2.10 | 2.46 | -0.36 | .0832 |
| G8 Uncooperativeness | 3.00 | 2.81 | 2.88 | 2.63 | 0.25 | .3026 |
| G9 Unusual thought content | 3.18 | 3.10 | 2.79 | 2.67 | 0.12 | .5445 |
| G10 Disorientation | 1.98 | 2.17 | 2.08 | 2.05 | 0.03 | .8413 |
| G11 Poor attention | 2.94 | 3.17 | 2.79 | 2.76 | 0.03 | .8889 |
| G12 Lack of judgement and insight | 3.96 | 3.71 | 3.49 | 3.56 | -0.07 | .6833 |
| G13 Disturbance of volition | 3.27 | 3.31 | 3.15 | 3.09 | 0.06 | .7098 |
| G14 Poor impulse control | 3.02 | 2.90 | 2.85 | 2.45 | 0.4 | **.0397** |
| G15 Preoccupation | 3.22 | 2.98 | 2.76 | 2.76 | -0.01 | .9762 |
| G16 Active social avoidance | 3.43 | 3.42 | 3.03 | 3.28 | -0.24 | .2291 |

PANSS-30 ES: 0.33, PANSS-6 ES: 0.51.

**Supplementary Table 2.** Study R092670-SCH-201, paliperidone 50mg eq. (n=78) vs placebo (n=82). Effect sizes for individual items.

|  | **Baseline score** | | **Endpoint score** | |  |  |
| --- | --- | --- | --- | --- | --- | --- |
| **Item** | **Placebo** | **Active** | **Placebo** | **Active** | **ES** | **p** |
| P1 Delusions | 3.84 | 3.79 | 3.61 | 2.94 | 0.67 | **.0037** |
| P2 Conceptual disorganization | 3.73 | 3.88 | 3.61 | 3.11 | 0.50 | **.0048** |
| P3 Hallucinatory behaviour | 3.27 | 3.47 | 3.18 | 2.57 | 0.62 | **.0066** |
| P4 Excitement | 2.59 | 2.86 | 2.99 | 2.41 | 0.58 | **.0049** |
| P5 Grandiosity | 2.04 | 2.38 | 2.30 | 1.87 | 0.43 | **.0116** |
| P6 Suspiciousness/persecution | 3.98 | 3.72 | 3.55 | 3.04 | 0.50 | **.0178** |
| P7 Hostility | 2.21 | 2.14 | 2.74 | 2.03 | 0.71 | **.0008** |
| N1 Blunted affect | 3.95 | 3.77 | 3.70 | 3.28 | 0.42 | **.0036** |
| N2 Emotional withdrawal | 3.94 | 3.76 | 3.76 | 3.24 | 0.51 | **.0013** |
| N3 Poor rapport | 3.49 | 3.14 | 3.32 | 2.85 | 0.47 | **.0089** |
| N4 Passive/apathetic, social withdrawal | 4.01 | 3.69 | 3.66 | 3.29 | 0.37 | **.0211** |
| N5 Difficulty in abstract thinking | 4.16 | 4.15 | 4.07 | 3.64 | 0.43 | **.0047** |
| N6 Lack of spontaneity and flow of conversation | 3.66 | 3.22 | 3.31 | 3.11 | 0.21 | .2504 |
| N7 Stereotyped thinking | 3.51 | 3.42 | 3.70 | 3.09 | 0.61 | **.0001** |
| G1 Somatic concern | 2.49 | 2.47 | 2.53 | 2.09 | 0.44 | **.0113** |
| G2 Anxiety | 3.17 | 3.15 | 3.24 | 2.61 | 0.64 | **.0009** |
| G3 Guilt feelings | 1.93 | 1.97 | 1.84 | 1.81 | 0.04 | .7753 |
| G4 Tension | 3.30 | 3.22 | 3.38 | 2.70 | 0.67 | **.0004** |
| G5 Mannerisms and posturing | 2.74 | 2.92 | 2.90 | 2.53 | 0.37 | **.0325** |
| G6 Depression | 2.35 | 2.55 | 2.32 | 1.91 | 0.40 | **.0133** |
| G7 Motor retardation | 2.45 | 2.41 | 2.13 | 1.93 | 0.19 | .2095 |
| G8 Uncooperativeness | 2.10 | 2.08 | 2.84 | 2.11 | 0.73 | **.0021** |
| G9 Unusual thought content | 3.17 | 3.28 | 3.32 | 2.73 | 0.58 | **.0008** |
| G10 Disorientation | 2.12 | 1.91 | 2.08 | 1.83 | 0.24 | .0763 |
| G11 Poor attention | 3.34 | 3.36 | 3.32 | 2.84 | 0.48 | **.0031** |
| G12 Lack of judgement and insight | 3.90 | 3.77 | 4.06 | 3.51 | 0.55 | **.0003** |
| G13 Disturbance of volition | 3.38 | 3.21 | 3.00 | 2.85 | 0.15 | .3654 |
| G14 Poor impulse control | 2.45 | 2.38 | 2.77 | 2.36 | 0.41 | **.0422** |
| G15 Preoccupation | 3.57 | 3.63 | 3.80 | 3.09 | 0.71 | **.0002** |
| G16 Active social avoidance | 3.48 | 3.42 | 3.57 | 2.95 | 0.62 | **.0005** |

PANSS-30 ES: 0.66, PANSS-6 ES: 0.52.

**Supplementary Table 3.** Study R076477-SCH-4012, paliperidone 6mg. (n=70) vs placebo (n=65). Effect sizes for individual items.

|  | **Baseline score** | | **Endpoint score** | |  |  |
| --- | --- | --- | --- | --- | --- | --- |
| **Item** | **Placebo** | **Active** | **Placebo** | **Active** | **ES** | **p** |
| P1 Delusions | 4.29 | 4.19 | 3.54 | 3.35 | 0.19 | .3976 |
| P2 Conceptual disorganization | 3.52 | 3.31 | 3.03 | 2.96 | 0.08 | .7190 |
| P3 Hallucinatory behaviour | 3.98 | 3.90 | 3.36 | 3.00 | 0.36 | .1612 |
| P4 Excitement | 2.86 | 2.43 | 2.36 | 2.27 | 0.09 | .6678 |
| P5 Grandiosity | 2.54 | 2.33 | 2.13 | 2.09 | 0.04 | .8194 |
| P6 Suspiciousness/persecution | 4.29 | 4.13 | 3.46 | 3.30 | 0.17 | .5027 |
| P7 Hostility | 2.75 | 2.46 | 2.45 | 1.98 | 0.47 | **.0258** |
| N1 Blunted affect | 3.35 | 3.64 | 3.11 | 2.92 | 0.19 | .2211 |
| N2 Emotional withdrawal | 3.62 | 3.64 | 3.07 | 3.02 | 0.05 | .8260 |
| N3 Poor rapport | 2.85 | 2.97 | 2.49 | 2.51 | -0.02 | .9262 |
| N4 Passive/apathetic, social withdrawal | 3.66 | 3.77 | 3.18 | 3.06 | 0.12 | .5410 |
| N5 Difficulty in abstract thinking | 4.11 | 4.03 | 3.65 | 3.55 | 0.09 | .6055 |
| N6 Lack of spontaneity and flow of conversation | 3.15 | 3.14 | 2.78 | 2.53 | 0.25 | .2304 |
| N7 Stereotyped thinking | 3.18 | 3.03 | 2.73 | 2.72 | 0 | .9860 |
| G1 Somatic concern | 2.65 | 2.63 | 2.26 | 2.09 | 0.17 | .3580 |
| G2 Anxiety | 3.37 | 3.23 | 2.55 | 2.86 | -0.31 | .1444 |
| G3 Guilt feelings | 1.97 | 2.03 | 1.77 | 1.57 | 0.20 | .1646 |
| G4 Tension | 2.97 | 2.9 | 2.43 | 2.42 | 0.01 | .9475 |
| G5 Mannerisms and posturing | 2.02 | 2.2 | 1.78 | 1.84 | -0.06 | .6761 |
| G6 Depression | 2.58 | 2.73 | 2.07 | 2.13 | -0.06 | .7564 |
| G7 Motor retardation | 2.35 | 2.66 | 1.98 | 2.06 | -0.09 | .5698 |
| G8 Uncooperativeness | 2.17 | 2.24 | 1.97 | 2.02 | -0.05 | .8129 |
| G9 Unusual thought content | 3.43 | 3.44 | 3.00 | 3.01 | -0.01 | .9725 |
| G10 Disorientation | 2.12 | 2.31 | 1.79 | 1.81 | -0.02 | .8929 |
| G11 Poor attention | 2.95 | 2.73 | 2.27 | 2.43 | -0.16 | .3861 |
| G12 Lack of judgement and insight | 3.37 | 3.63 | 3.16 | 3.01 | 0.15 | .4995 |
| G13 Disturbance of volition | 2.89 | 2.71 | 2.42 | 2.50 | -0.08 | .6655 |
| G14 Poor impulse control | 2.62 | 2.41 | 2.24 | 2.01 | 0.23 | .3039 |
| G15 Preoccupation | 3.38 | 3.43 | 3.15 | 2.89 | 0.26 | .2269 |
| G16 Active social avoidance | 3.62 | 3.60 | 2.96 | 3.02 | -0.05 | .7928 |

PANSS-30 ES: 0.08, PANSS-6 ES: 0.20.

**Supplementary Table 4.** Study RIS-INT-3, risperidone 6mg. (n=86) vs placebo (n=88). Effect sizes for individual items.

|  | **Baseline score** | | **Endpoint score** | |  |  |
| --- | --- | --- | --- | --- | --- | --- |
| **Item** | **Placebo** | **Active** | **Placebo** | **Active** | **ES** | **p** |
| P1 Delusions | 4.15 | 4.22 | 4.28 | 3.38 | 0.90 | **<.0001** |
| P2 Conceptual disorganization | 3.72 | 3.86 | 3.91 | 3.05 | 0.86 | **<.0001** |
| P3 Hallucinatory behaviour | 3.88 | 3.86 | 4.04 | 2.78 | 1.26 | **<.0001** |
| P4 Excitement | 2.77 | 2.67 | 2.92 | 1.90 | 1.02 | **<.0001** |
| P5 Grandiosity | 2.30 | 2.84 | 2.48 | 2.17 | 0.31 | .1112 |
| P6 Suspiciousness/persecution | 3.97 | 3.82 | 4.03 | 3.03 | 1.00 | **<.0001** |
| P7 Hostility | 2.53 | 2.38 | 3.01 | 1.81 | 1.20 | **<.0001** |
| N1 Blunted affect | 3.59 | 3.87 | 3.46 | 3.12 | 0.34 | **.0384** |
| N2 Emotional withdrawal | 3.47 | 3.63 | 3.56 | 3.17 | 0.40 | .0302 |
| N3 Poor rapport | 3.01 | 3.34 | 3.25 | 2.71 | 0.54 | **.0119** |
| N4 Passive/apathetic, social withdrawal | 3.56 | 3.67 | 3.61 | 3.03 | 0.58 | **.0014** |
| N5 Difficulty in abstract thinking | 3.85 | 4.09 | 3.99 | 3.48 | 0.51 | **.0104** |
| N6 Lack of spontaneity and flow of conversation | 2.95 | 3.35 | 3.25 | 2.82 | 0.43 | **.0484** |
| N7 Stereotyped thinking | 3.43 | 3.52 | 3.36 | 2.86 | 0.50 | **.0088** |
| G1 Somatic concern | 2.89 | 2.83 | 2.90 | 2.41 | 0.49 | **.0179** |
| G2 Anxiety | 3.25 | 3.03 | 3.20 | 2.4 | 0.80 | **<.0001** |
| G3 Guilt feelings | 2.11 | 2.06 | 1.92 | 1.68 | 0.24 | .1340 |
| G4 Tension | 3.05 | 2.93 | 3.12 | 2.14 | 0.98 | **<.0001** |
| G5 Mannerisms and posturing | 2.69 | 2.77 | 2.85 | 2.22 | 0.63 | **.0005** |
| G6 Depression | 2.72 | 2.33 | 2.27 | 2.14 | 0.13 | .4749 |
| G7 Motor retardation | 2.08 | 2.3 | 2.02 | 2.07 | -0.05 | .7653 |
| G8 Uncooperativeness | 2.17 | 2.21 | 2.72 | 1.81 | 0.91 | **.0001** |
| G9 Unusual thought content | 3.93 | 4.19 | 4.15 | 3.27 | 0.89 | **<.0001** |
| G10 Disorientation | 1.94 | 1.95 | 2.13 | 1.50 | 0.63 | **<.0001** |
| G11 Poor attention | 2.93 | 2.92 | 3.09 | 2.15 | 0.93 | **<.0001** |
| G12 Lack of judgement and insight | 3.83 | 3.80 | 4.00 | 3.39 | 0.61 | **.0006** |
| G13 Disturbance of volition | 2.81 | 3.05 | 2.89 | 2.28 | 0.61 | **.0021** |
| G14 Poor impulse control | 2.32 | 2.40 | 2.76 | 1.68 | 1.08 | **<.0001** |
| G15 Preoccupation | 3.56 | 3.81 | 3.83 | 2.78 | 1.04 | **<.0001** |
| G16 Active social avoidance | 3.23 | 3.21 | 3.43 | 2.57 | 0.86 | **<.0001** |

PANSS-30 ES: 0.94, PANSS-6 ES: 0.83.

**Supplementary Table 5.** Study RIS-USA-121, RIS DPT 25mg eq. (n=105) vs placebo (n=107). Effect sizes for individual items.

|  | **Baseline score** | | **Endpoint score** | |  |  |
| --- | --- | --- | --- | --- | --- | --- |
| **Item** | **Placebo** | **Active** | **Placebo** | **Active** | **ES** | **p** |
| P1 Delusions | 3.75 | 3.57 | 3.76 | 3.24 | 0.52 | **.0028** |
| P2 Conceptual disorganization | 3.15 | 2.97 | 3.30 | 2.75 | 0.55 | **.0011** |
| P3 Hallucinatory behaviour | 3.06 | 3.25 | 3.24 | 3.08 | 0.15 | .4121 |
| P4 Excitement | 2.35 | 2.06 | 2.55 | 2.23 | 0.32 | .0655 |
| P5 Grandiosity | 2.41 | 2.31 | 2.31 | 2.01 | 0.30 | .0772 |
| P6 Suspiciousness/persecution | 3.64 | 3.70 | 3.70 | 3.32 | 0.39 | **.0249** |
| P7 Hostility | 1.82 | 1.70 | 2.09 | 1.93 | 0.15 | .3642 |
| N1 Blunted affect | 3.08 | 3.12 | 2.98 | 2.62 | 0.37 | **.0121** |
| N2 Emotional withdrawal | 3.21 | 3.32 | 3.45 | 2.80 | 0.65 | **<.0001** |
| N3 Poor rapport | 2.39 | 2.45 | 2.65 | 2.15 | 0.50 | **.0010** |
| N4 Passive/apathetic, social withdrawal | 3.22 | 3.30 | 3.37 | 2.78 | 0.59 | **.0001** |
| N5 Difficulty in abstract thinking | 3.93 | 3.79 | 3.80 | 3.64 | 0.16 | .3040 |
| N6 Lack of spontaneity and flow of conversation | 2.67 | 2.96 | 2.98 | 2.42 | 0.55 | **.0003** |
| N7 Stereotyped thinking | 2.63 | 2.52 | 2.79 | 2.44 | 0.34 | **.0093** |
| G1 Somatic concern | 2.47 | 2.63 | 2.37 | 2.29 | 0.08 | .6594 |
| G2 Anxiety | 3.22 | 3.11 | 3.14 | 2.87 | 0.27 | .0915 |
| G3 Guilt feelings | 2.39 | 2.27 | 2.06 | 1.97 | 0.09 | .5545 |
| G4 Tension | 2.67 | 2.64 | 2.67 | 2.40 | 0.27 | .0623 |
| G5 Mannerisms and posturing | 2.11 | 1.94 | 2.04 | 1.75 | 0.30 | **.0157** |
| G6 Depression | 2.50 | 2.83 | 2.66 | 2.52 | 0.14 | .4016 |
| G7 Motor retardation | 2.18 | 2.21 | 1.97 | 1.89 | 0.08 | .5215 |
| G8 Uncooperativeness | 1.70 | 1.50 | 1.99 | 1.91 | 0.08 | .6164 |
| G9 Unusual thought content | 3.20 | 3.08 | 3.18 | 3.06 | 0.12 | .4320 |
| G10 Disorientation | 1.79 | 2.17 | 2.14 | 1.80 | 0.34 | **.0033** |
| G11 Poor attention | 2.64 | 2.44 | 2.68 | 2.37 | 0.31 | .0535 |
| G12 Lack of judgement and insight | 3.59 | 3.61 | 3.58 | 3.25 | 0.33 | **.0318** |
| G13 Disturbance of volition | 2.56 | 2.51 | 2.51 | 2.45 | 0.06 | .6629 |
| G14 Poor impulse control | 2.04 | 1.85 | 2.15 | 2.11 | 0.05 | .7737 |
| G15 Preoccupation | 2.99 | 2.89 | 3.14 | 2.69 | 0.46 | **.0083** |
| G16 Active social avoidance | 3.05 | 3.10 | 3.04 | 2.69 | 0.35 | **.0240** |

PANSS-30 ES: 0.52, PANSS-6 ES: 0.62.
